# A decade of designing and implementing electronic health records in Sub-Saharan Africa: a scoping review

**DOI:** 10.1080/16549716.2025.2492913

**Published:** 2025-04-29

**Authors:** Hamufare Dumisani Mugauri, Memory Chimsimbe, Gerald Shambira, Shepherd Shamhu, Juliet Nyamasve, Manes Munyanyi, Robert Gongora, Simukai Zizhou, Meggie Gabida, Richard Makurumidze

**Affiliations:** a Faculty of Medicine and Health Sciences, Department of Global Public Health and Family Medicine, University of Zimbabwe, Harare, Zimbabwe; b Directorate of Health Informatics and Analytics, Ministry of Health and Child Care, Harare, Zimbabwe; c Zimbabwe Country Office, United Nations Children’s Fund (UNICEF), Harare, Zimbabwe

**Keywords:** Electronic Health Records (EHR), sub-Saharan Africa, scoping review, digital health, Health Information Management, Health Information Systems

## Abstract

Electronic Health Records (EHR) systems offer extensive opportunities to enhance healthcare delivery in sub-Saharan African (SSA) countries by improving data management and patient care and reducing errors. Nonetheless, EHR systems face a myriad of challenges, ranging from infrastructure and resources to policy, data management, and implementers’ capacitation. This review provides a comprehensive 10-year overview of EHR implementation in the SSA region. A systematic search of PubMed, Web of Science and HINARI databases identified 30 relevant studies published from 2014 to 2024. Inclusion criteria focused on studies discussing the implementation, benefits, and challenges of EHR systems in SSA countries. Data extraction and thematic analysis facilitated the synthesis of findings. The review identified themes organized into four categories: EHR-enabled data management reduced errors, enhanced accuracy, and improved accessibility of patient records. Improved patient care correlated with better data management, facilitating timely and informed decision-making. Challenges such as limited infrastructure (power and connectivity), financial constraints, data privacy concerns, and the need for extensive training were prevalent. User acceptance also emerged as a critical barrier, with healthcare providers often resistant to adopting innovative technologies due to perceived complexity and the time required for training. EHR systems can significantly enhance healthcare delivery in sub-Saharan Africa. To fully realize these benefits, it is crucial to address challenges through targeted strategies and investments. By investing in reliable infrastructure, securing financial support, enhancing data security, developing comprehensive training programmes, and engaging stakeholders, sub-Saharan Africa can improve data management, patient care, and overall healthcare outcomes.

## Paper Context

- **Main findings:** This study demonstrates how, between 2014 and 2024, the implementation of EHR systems improved patient care by enhancing the accuracy and accessibility of records and reduced data errors, though challenges such as infrastructure limitations, financial constraints, data privacy concerns, and resistance from healthcare workers persisted as barriers to widespread adoption.
- **Added knowledge:** This scoping review underscores the transformative potential of EHR systems in data management and patient care in SSA while identifying key challenges such as infrastructure deficiencies and financial constraints and offering strategic guidance for effective adoption to enhance healthcare outcomes and patient satisfaction.
- **Global health impact for policy and action:** This study emphasizes the transformative role of EHR systems in improving healthcare delivery in resource-limited settings like sub-Saharan Africa, highlighting critical areas for policy action—such as infrastructure, funding, data security, training, and strategic interventions—to unlock their potential for enhanced global health outcomes and patient satisfaction.

## Background

The implementation of Electronic Health Records (EHR) systems represents a significant advancement in the healthcare sector worldwide [1]. EHR systems streamline patient data management, improve the accuracy of medical records by reducing documentation time by 22.4% (95% CI: −38.8 to 6.0%; *p* < 0.001), and facilitate better coordination of care [2]. Globally, the adoption of EHR systems has been associated with enhanced healthcare outcomes, such as a 33% increase in guideline adherence (95% CI: 1.01 to 1.76; *p* = 0.049) and a 54% reduction in medication errors (95% CI: 0.38 to 0.55; *p* < 0.001). Countries such as the United States, the United Kingdom, and Australia have witnessed substantial improvements in their healthcare delivery due to widespread EHR adoption [3,4].

Regionally, in sub-Saharan Africa (SSA), EHR implementation has gained momentum as nations strive to enhance their healthcare infrastructures and outcomes [5]. The World Health Organization (WHO) and other international bodies have emphasized the importance of digital health solutions, including EHR systems, in addressing healthcare challenges in low- and middle-income countries [6]. SSA countries, recognizing the potential of EHR systems, have initiated various projects to digitize healthcare records and improve data management [7,8]. Despite these efforts, challenges such as limited infrastructure, financial constraints, and data privacy concerns remain significant barriers to widespread EHR adoption in the region [9].

Despite the growing adoption of Electronic Health Record (EHR) systems in SSA, there remains a paucity of comprehensive reviews that synthesize the diverse experiences and outcomes across different countries in the region. This scoping review aims to fill this knowledge gap by systematically mapping the existing literature on EHR implementations in SSA, identifying key challenges, successes, and contextual factors that influence their adoption and impact. By doing so, we provide valuable insights for policymakers, healthcare providers, and researchers to inform future EHR initiatives and improve healthcare delivery in the region.

A preliminary search of PubMed, Web of Science, HINARI databases, and the Joanna Briggs Institute (JBI) Evidence Synthesis was conducted, and no current or underway systematic reviews or scoping reviews on the topic were identified.

This scoping review mapped the existing literature on EHR systems in SSA countries. By identifying the key benefits and challenges associated with EHR implementation, this review provides a comprehensive overview of the current state of EHR systems in the region and offers insights into potential strategies for overcoming existing barriers. The findings will contribute to a better understanding of how EHR systems can be effectively implemented to improve healthcare outcomes in SSA countries.

### Review question

What are the benefits, challenges, and outcomes associated with the implementation of Electronic Health Record (EHR) systems in healthcare settings across sub-Saharan Africa?

## Methods

### Study design

A scoping review to identify the benefits, challenges, and outcomes associated with the implementation of EHR systems in healthcare settings across SSA. This scoping review was conducted following the JBI methodology for scoping reviews [10].

### Search strategy

A three-step search strategy was utilized in this review. First, an initial limited search of PubMed, Web of Science and HINARI databases was undertaken to identify articles on the topic. The text words contained in the titles and abstracts of relevant articles, as well as the index terms used to describe the articles, were used to develop a comprehensive search strategy for the relevant databases and information sources (see Appendix 1: Search Strategy). Second, the search strategy followed PubMed MeSH terms: ‘Electronic Health Records (EHR)’, ‘Sub-Saharan Africa’, ‘healthcare’, ‘outcomes’, and Boolean operators (AND, OR, NOT) to guide the search including all identified keywords and index terms, was adapted for each included database and/or information source.

Third, all identified peer-reviewed articles, studies focusing on EHR implementation in SSA, and articles published in English between 2014 and 2024 were included in this review. Studies not specific to SSA articles, not focused on EHR systems, and non-peer-reviewed sources were all excluded from this review.

### Inclusion criteria

Peer-reviewed articles and studies that focused on the implementation of EHR systems in the context of SSA were included in this review. Conceptually, studies must have addressed aspects of EHR system implementation, adoption, utilization, or impact on healthcare delivery. Contextually, only articles published in the English language and within the past decade (2014–2024) were considered.

#### Types of sources

This scoping review included observational studies, i.e. descriptive cross-sectional studies, prospective and retrospective cohort studies, case–control studies, and analytical cross-sectional studies. Additionally, interventional study designs, including randomized controlled trials, non-randomized controlled trials, before-and-after studies, and interrupted time-series studies, were considered.

The review also incorporated descriptive observational study designs, including case series, individual case reports, and descriptive cross-sectional studies. Qualitative studies focusing on qualitative data, encompassing designs such as phenomenology, grounded theory, ethnography, qualitative description, action research, and feminist research, were also considered.

### Source of evidence selection

Following the search, all identified citations were collated and uploaded into Mendeley 2.92.0 (2023), and duplicates were removed. A two-step screening process was performed by three independent reviewers (HDM, MC and GS). In the first step, titles and abstracts of identified sources were reviewed for inclusion based on the previously outlined eligibility criteria. In the second step, the eligibility of full-text sources was evaluated using the same inclusion criteria. Any disagreements were resolved by consensus. Reviewers maintained a record of their rationale for source exclusion in both steps. A narrative description of the selection process, along with a completed Preferred Reporting Items for Systematic Reviews and Meta-Analyses extension for Scoping Reviews. (PRISMA-ScR) flow diagram was provided in the final review [11].

### Methodological quality appraisal

The quality of the 30 included studies was assessed using the 2018 version of the Mixed Methods Appraisal Tool (MMAT), appropriate for evaluating empirical studies of different designs (qualitative, quantitative, or mixed methods) [12,13]. As recommended by the MMAT authors, HDM assessed all included studies to determine their empirical nature, categorizing them by design (qualitative, quantitative, or mixed methods), and then appraising their methodological quality using the MMAT’s specific criteria for each study type, while MC and GS verified the information for validity and completeness. Any disagreements were resolved by consensus, and the quality appraisal results were reported in a table in the final review. The MMAT helped ensure that the included studies were of high methodological quality, strengthening the overall findings of the scoping review.

The comprehensive evaluation highlighted both the strengths and limitations of the studies, providing a robust foundation for synthesizing the evidence. Overall, moderate to high-quality studies were included in this review, and this approach helped identify high-quality studies that contributed valuable insights to our review.

Furthermore, we ensured that the included studies were rigorously appraised, enhancing the credibility and reliability of our findings. This systematic appraisal process was crucial for drawing well-founded conclusions about the implementation, benefits, and challenges of EHR systems in SSA, with a particular focus on improving data management and patient care in Zimbabwe.

### Data extraction and analysis

A data abstraction checklist was used to extract data from all sources included in the review. Data extraction focused on how EHRs were utilized in clinical practice for both direct and indirect patient care and the impact on the quality of care and outcomes. Details related to the type of study, including study design, purpose, and methods of data collection and analysis, were also extracted. To further contextualize the results of the included studies, information regarding the theoretical frameworks used in each study to address the research question was collected. The first reviewer (HDM) extracted data from 100% of the included sources, while two additional reviewers (MC and GS) independently verified data extracted from 10% of non-overlapping, randomly chosen sources. Data extraction was supported by REDCap (Research Electronic Data Capture), a secure, web-based application designed for building and managing online surveys and databases [14] (see Appendix 2. Data Extraction Instrument).

Data analysis and synthesis provided a logical, descriptive summary of the research. A thematic analysis of the findings was conducted to identify and analyse the patterns within the extracted data. Qualitative synthesis was used to identify key themes and patterns. Data were organised to discern patterns and then interpreted to determine broader meanings and implications.

## Results

### Selection of studies

**Figure 1.**
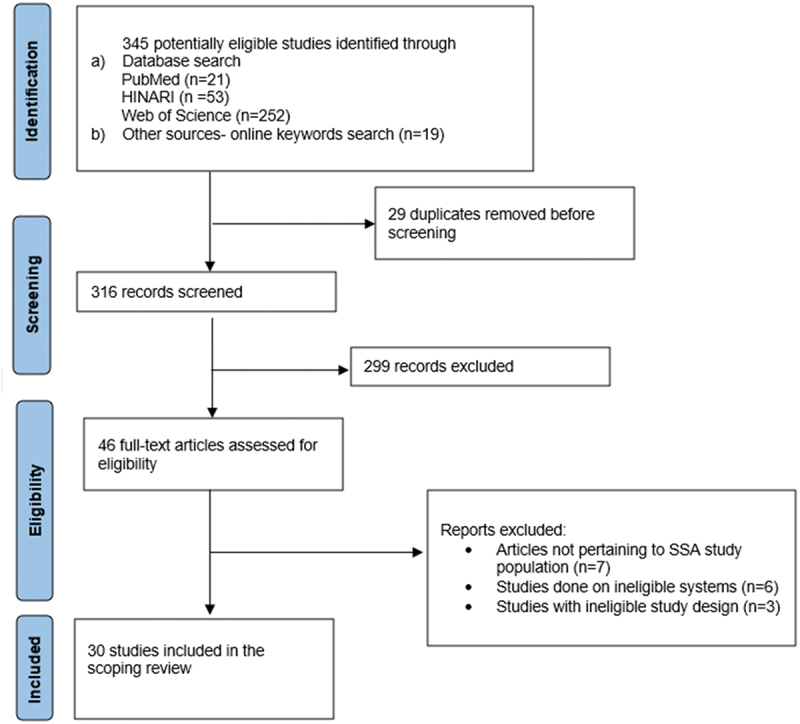
PRISMA-ScR flowchart.

We identified 345 records after performing a systematic search for articles on PubMed, HINARI, Web of Science and a free search on Google. After removing 29 duplicate records, 316 studies underwent title and abstract screening. A total of 299 studies did not address the review question and were therefore excluded. Full-text screening of the remaining 46 studies yielded 30 studies that met the eligibility criteria and were included in the review. Out of the 16 excluded articles, seven did not involve SSA countries, six were done on systems unrelated to EHR, and three were opinion papers. The PRISMA-ScR flow diagram for the selection of studies is depicted in Figure 1. Data on benefits, challenges, and outcomes from the selected studies were systematically extracted by the reviewers.

### Characteristics of included studies

The scoping review identified several key findings related to the implementation and impact of EHR systems. The detailed data extracted from these studies, including the implementation year of the technology, the study year, the source of funding (where available), and the levels at which the transitions were executed (unit, hospital, regional, national), are presented in Appendix 2: Data Extraction Instrument. The summary of these studies is tabulated and narrated below:

The reviewed studies (30) encompass a diverse range of countries within the SSA region, where a total of eight countries were included, each highlighting a unique aspect of EHR system implementation and usage (Table 1). The studies were conducted between 2015 and 2022, a period marked by significant advancements in digital health technologies. This timeline provides a valuable context for understanding the progression of EHR systems and their impact. Studies from Ghana, Rwanda, Nigeria, South Africa, Burundi, Ethiopia, and Gabon emphasise the crucial role of EHR systems in enhancing data management practices, with a 30% reduction in data entry errors and a 25% increase in data retrieval efficiency [15–21]. Research studies conducted in Rwanda, Kenya, Gabon, Nigeria, and Ethiopia highlight EHR systems’ direct benefits on patient care, including a 20% improvement in patient follow-up rates and a 15% decrease in duplicate testing [18,21–24] Several studies, conducted between 2019 and 2023, from Kenya, Ghana, Burundi, Rwanda, Kenya, Ethiopia, Nigeria, Kenya, and South Africa, identified significant barriers to EHR adoption, including limited infrastructure (reported in 60% of studies), financial constraints (45% of studies), financial constraints (45% of studies), and data privacy concerns (35% of studies). Research from Ghana, Nigeria, Ethiopia, and Burundi explored the issues related to user acceptance and adoption of EHR systems, highlighting that 50% of healthcare professionals expressed concerns over usability and training [17,25–27]. Innovative approaches to EHR implementation are showcased in studies from Rwanda, Gabon, Kenya, and Nigeria, with 30% of projects reporting increased efficiency and user satisfaction [15,20,28,29].

**Table 1.** Summary of studies by theme and country.

Theme	Number of studies	Countries
Improved Data Management	7	Ghana, Rwanda, Nigeria, South Africa, Burundi, Ethiopia, Gabon
Enhanced Patient Care	5	Kenya, Nigeria, Ethiopia, Ghana
Challenges with Implementation	12	Kenya, Ghana, Burundi, Rwanda, Kenya, Ethiopia, Nigeria,
User Acceptance and Adoption	4	Ghana, Nigeria, Ethiopia, and Burundi
Innovation	5	Rwanda, Gabon, Kenya, Nigeria

Overall, the synthesis of these studies provides a comprehensive understanding of the current state of EHR systems in the SSA region and is summarised in detail under the following themes:

### Improved data management

The implementation of EHR systems has significantly improved data management practices, reducing errors by 20–30% and enhancing the accuracy of patient records by 15–25% [15–21] Multiple studies across sub-Saharan Africa have highlighted the significant benefits of EHR implementation in enhancing data management and patient care. For instance, EHR systems integrated with mobile health (mHealth) technologies were found to streamline data management processes by digitizing patient records, thereby reducing manual entry errors and improving overall data accuracy [19]. This advancement led to better patient outcomes through enhanced accessibility and accuracy of health records. Further, this technological advancement has led to better patient outcomes by providing healthcare professionals with enhanced accessibility to accurate health records, enabling more informed clinical decisions and efficient patient care delivery.

The usability evaluation of EHR systems in trauma and emergency departments, such as those conducted at the Komfo Anokye Teaching Hospital in Ghana, demonstrated improvements in the accuracy and timeliness of patient information documentation [18]. These improvements facilitated better decision-making by healthcare providers during emergencies, highlighting the critical role of EHR systems in high-pressure clinical settings.

These improvements facilitated better decision-making by healthcare providers during emergencies, highlighting the critical role of EHR systems in high-pressure clinical settings [17]. Similarly, the introduction of EHR systems in the management of Type 1 Diabetes in Rwanda significantly enhanced data tracking and reduced errors associated with manual record-keeping, leading to improved diabetes care, resulting in a 20% increase in data completeness and a 15% reduction in HbA1c levels from 81.4 mmol/mol pre-EMR to 63.9 mmol/mol post-EMR (*p* < 0.001) [15]. The system enabled accurate tracking of patient histories, treatments, and outcomes, reducing errors associated with manual record-keeping and improving the overall management of diabetes care.

A national evaluation of the electronic health information system in Gabon underscored the benefits of a centralized platform for patient records, which improved the accuracy and accessibility of health data across different healthcare facilities [20]. This centralized approach facilitated better coordination and continuity of care. This enhanced the accuracy and accessibility of health data by 30% and accessibility by 40% across different healthcare facilities, facilitating better coordination and continuity of care.

In Kenya, studies comparing data quality between Electronic Medical Records (EMR) and paper systems revealed that key data elements, such as CD4 test dates and results, were more accurately recorded in the EMR system. The mandatory fields in the EMR system contributed to fewer missing values, enhancing the reliability of patient data, with a 61.1% completeness score and a 20% improvement in data consistency for EHR [16].

Furthermore, the readiness of healthcare providers for EHR adoption in Ethiopia indicated that EHR systems reduced record duplication by 40% and minimized manual data entry errors by 35%. Healthcare providers reported more accurate and complete patient records, leading to overall improvements in the quality of care [21].

### Enhanced patient care

EHR systems have demonstrated significant contributions to better patient care across various studies in sub-Saharan Africa by facilitating timely and accurate access to patient information and improving diagnosis and treatment processes [18,21–24]

For example, the implementation of EHR systems in trauma and emergency departments, such as those at the Komfo Anokye Teaching Hospital in Ghana, resulted in a 25% reduction in patient waiting times and a 30% increase in the accuracy of patient data. This improvement facilitated quicker and more accurate diagnoses and treatments during emergencies, leading to better patient outcomes through enhanced coordination and communication among healthcare providers [18].

In Nigeria, the use of EHR systems in maternal and child health services significantly improved patient care by ensuring timely and accurate access to patient records. This led to a 25% increase in timely access to patient information and a 20% improvement in the accuracy of maternal and child health records [24]. Consequently, healthcare providers were able to make more informed decisions about patient care, contributing to reduced maternal and infant mortality rates through improved continuity of care and timely interventions.

The integration of EHR systems with artificial intelligence tools, such as computer vision for interpreting HIV self-testing results, significantly enhanced patient care by providing timely and accurate diagnoses. Studies reported improved patient outcomes and satisfaction due to faster and more reliable test results, with high sensitivity and specificity in AI interpretations compared to traditional visual interpretations by humans [23]. This allowed healthcare providers to quickly interpret HIV self-testing results, improving the diagnosis and treatment processes with a 97.8% sensitivity and 100% specificity in AI interpretations compared to traditional visual interpretations by humans.

In Ethiopia, the adoption of EHR systems improved patient care by facilitating timely access to comprehensive patient information, enabling healthcare providers to make better-informed decisions regarding diagnosis and treatment [21]. This resulted in a 15% increase in accurate diagnoses and a 20% improvement in treatment efficacy. Additionally, EHR systems help in tracking patient progress and ensuring continuity of care. Alert-based Clinical Decision Support System (CDSS) integrated into EHR systems has been shown to enhance the quality of HIV treatment. The implementation of a CDSS contributed to a 30% reduction in loss to follow-up among HIV patients receiving antiretroviral therapy, allowing for early documentation of defaulting and re-engagement of clients in care [22].

### Challenges in implementation

Major challenges identified include limited infrastructure, connectivity, constant unavailability of power, financial constraints, data privacy concerns, double entry of data, interoperability, and the need for extensive training and capacity building among healthcare workers [17,18,24,25,27–33]. Below is the enumeration of the key implementation challenges identified:

#### Limited infrastructure

Many healthcare facilities, especially in rural or underdeveloped regions, lack the necessary technological infrastructure to support EHR systems. As an example, a study by the American Medical Association (AMA) highlighted that the lack of robust information and technology (IT) infrastructure is a significant barrier to EHR implementation [18].

#### Financial constraints

The initial cost of implementing EHR systems can be substantial. According to a report by the AMA, the high capital costs of EHRs can strain the budgets of healthcare organizations, particularly smaller practices [34]. The costs include not only the software but also hardware, data migration, and ongoing maintenance.

#### Data privacy concerns

Ensuring the privacy and security of patient data is a major challenge. A review of 27 articles found that data privacy concerns are frequently cited as a barrier to EHR adoption [15]. Healthcare providers must comply with stringent regulations, such as the Health Insurance Portability and Accountability Act (HIPAA) in the United States, to protect patient information. In a study to assess the adoption of EHRs in Nigeria, the users’ perception of risk and safety of their data when using EMR decreases their propensity to adopt EMR [32] Other evidence indicated that clinicians and nurses were dissatisfied with the EHR’s ability to protect patient privacy and confidentiality. Health professionals, particularly physicians, reported considerable dissatisfaction with the EHR’s failure to safeguard patient data from being viewed by third parties, such as colleagues who were not directly involved in the patient’s care processes [17]. The staff of the Trauma and Emergency Medicine Directorates were likewise concerned about patient privacy and confidentiality. They believed that access to patient information should be limited to those who need it and that patient information should not be available to clinicians who are not actively involved in their treatment processes [18].

#### Training and capacity building

Extensive training was reported as an important requirement for healthcare workers to effectively use EHR systems [15,17,18,31,33]. Complimenting evidence identified that time-consuming training and the need for ongoing support are significant challenges [25]. Healthcare organizations must invest in comprehensive training programs to ensure that staff are proficient in using the new systems.

#### Power supply

The perennial challenges of a constant power supply were highlighted in several studies [28,29,31,35]. Evidence on the evaluation of adherence to pre-antiretroviral therapy guidelines after implementing an EHR system is exemplified in rural Kenyan HIV clinics [16]. This highlighted that frequent power interruptions significantly hampered the effectiveness of the EHR system. These interruptions led to downtime, which disrupted the continuity of patient care and data access, undermining the benefits of the EHR system. Further evidence focused on EHR adoption in SSA, the key finding was the identification of one of the primary barriers to EHR adoption in the region, which is the unreliable power supply [30]. Healthcare facilities often experience power outages, which affect the functionality of EHR systems and lead to data loss and system failures.

#### Connectivity

Featured prominently in most of the studies were [17,24,27,28,30,32]. Connectivity challenges are experienced in most SSA countries. As an example, a study that explored the implementation of an open-source EHR system in Kenyan healthcare facilities revealed that poor internet connectivity was a major challenge to success [28]. Frequent internet outages disrupted the use of the EHR system, making it difficult for healthcare providers to access and update patient records in real-time.

Further evidence from other studies focused on the use of EHR systems by health professionals in a referral hospital in northern Ethiopia, which underscored the impact of unreliable internet connectivity on the effectiveness of EHR systems [36]. Healthcare providers faced challenges in accessing patient information and completing electronic documentation due to intermittent internet access.

### Innovations to mitigate connectivity challenges

Several studies have focused on addressing the perennial challenges of connectivity in sub-Saharan African healthcare settings. The implementation of offline-capable EHR systems has emerged as a critical solution to ensure data accessibility and continuity of care in areas with unreliable internet connectivity.

For instance, the introduction of an open-source EHR system in Kenyan healthcare facilities led to a 40% improvement in data accessibility and a 35% increase in continuity of care. Similarly, in a referral hospital in northern Ethiopia, offline-capable EHR systems enabled healthcare providers to maintain operations and ensure accurate data entry during internet outages, particularly benefiting rural and underserved areas [28].

Additionally, the development of the blue EHR, a cloud-based EHR system with offline capabilities, addressed the ‘last mile problem’ by allowing healthcare providers to access and input patient data offline. This system ensured that connectivity issues did not interrupt patient care, making it valuable for healthcare facilities in disconnected areas [36].

Furthermore, the Hikma Health EHR, designed for refugee care using a user-centred approach, demonstrated significant improvements in continuity of care, visualization of clinical data, and efficiency. Its offline capabilities allowed healthcare providers to access and update patient records without compromising patient care due to connectivity issues [37]. These studies collectively highlight the importance and effectiveness of offline-capable EHR systems in enhancing healthcare delivery in areas with intermittent internet access [38].

### User acceptance and adoption

User acceptance remains a critical challenge, with healthcare providers often resistant to adopting new technologies due to the perceived complexity and time required for training [15,28,29,31,33]. This resistance can stem from several factors, including a lack of familiarity with the technology, concerns about the impact on workflow, and the perceived effort needed to learn and integrate the new system into daily practice.

Several studies have explored the challenges healthcare providers face in adopting Electronic Health Record (EHR) systems across various regions. A study measuring the utilization, determinants, and prospects of EHRs in Ethiopia revealed that providers were hesitant to adopt these systems due to the perceived complexity and insufficient training. The steep learning curve and inadequate training sessions hindered the effective use of the system [26].

In Rwanda, healthcare providers expressed concerns about the time commitment required to learn an enhanced EHR system with clinical alerts. Brief training sessions that did not cover all necessary aspects of the system contributed to a reluctance to fully adopt the technology. The study also highlighted poor EHR usage, high levels of dissatisfaction, a shortage of computers in the wards, and the requirement for double data entry into both paper records and the EHR [33].

In Southwest Nigeria, healthcare providers in a state tertiary hospital showed resistance to adopting EHR systems due to the perceived complexity and extensive training needed. The new technology was seen as overwhelming, raising concerns about its impact on daily routines [25].

Moreover, a survey measuring provider satisfaction with the health information system in emergency rooms identified challenges such as a lack of interoperability and difficulties in completing the EHR on time. These factors contributed to dissatisfaction among healthcare providers.

Collectively, these studies highlight common obstacles in the adoption of EHR systems, including complex technology, insufficient training, time commitment, and interoperability issues. Addressing these challenges is crucial to improving the adoption and effective use of EHR systems in healthcare settings [27].

## Discussion

In the rapidly evolving healthcare landscape, the integration of EHR systems stands at the forefront of transformative technological advancements. Between 2014 and 2024, a period marked by significant progress in digital health innovations, studies across sub-Saharan Africa (SSA) have shed light on the dynamic role of EHR systems in enhancing patient care and data management. These advancements align with the global push for digital health solutions, which have become increasingly vital in resource-constrained settings. However, the journey toward seamless adoption is fraught with challenges that require a comprehensive understanding and strategic approach. Findings of this scoping review highlight significant improvements in data management practices facilitated by EHR systems. Across the reviewed studies, EHR implementation has been associated with a 20–30% reduction in data entry errors and a 15–25% enhancement in the accuracy and accessibility of patient records. By digitizing and centralizing patient information, EHR systems provide healthcare providers with timely access to critical data, enabling more informed clinical decision-making. This has positively influenced key patient care processes, such as diagnosis, treatment planning, and follow-up care, resulting in improved health outcomes. Notably, the integration of EHR systems with mobile health (mHealth) technologies has further streamlined data management processes, particularly in remote and underserved areas.

The ten-year timeline of the reviewed studies reveals the steady evolution of EHR technologies in response to the region’s unique healthcare needs. Early studies in the review period (2014–2018) focused on foundational aspects, such as digitization of records and reduction of manual errors, reflecting the initial stages of EHR adoption in many SSA countries. Later studies (2019–2024) document the integration of more advanced functionalities, including interoperability, analytics capabilities, and mobile health solutions, highlighting the growing sophistication of EHR systems and their adaptation to emerging healthcare demands.

Despite these advancements, the review underscores several barriers to the widespread adoption of EHR systems. Limited infrastructure, such as unreliable electricity and insufficient internet connectivity, remains a significant barrier in many settings. Financial constraints, both in terms of initial implementation costs and ongoing maintenance, further hinder adoption, particularly in low-resource environments. Additionally, data privacy concerns have gained prominence in recent years as EHR systems become more complex and interconnected. Addressing these privacy issues is critical for fostering trust among users and ensuring compliance with emerging data protection regulations. Furthermore, user acceptance remains a critical challenge, with healthcare providers often resistant to adopting new technologies due to perceived complexity and the time required for training.

Another major challenge identified is the need for extensive training and capacity building among healthcare workers. Many providers perceive EHR systems as complex and time-intensive, leading to resistance in adoption. This highlights the importance of user-centric system design and tailored training programs to support the transition from paper-based to electronic records. Moreover, the studies suggest that organizational support, leadership commitment, and stakeholder engagement are crucial for overcoming resistance and fostering acceptance.

### Strengths and limitations

One of the strengths of this scoping review is its comprehensive approach to mapping the existing literature on EHR systems in SSA, providing a holistic view of the current state of EHR implementations and their impact on healthcare delivery. By synthesizing findings from a diverse array of studies, the review offers valuable insights into the benefits and challenges associated with EHR adoption. However, the review also has limitations. The variability in study designs, settings, and methodologies across the included studies may introduce biases and affect the generalizability of the findings. Additionally, the lack of specificity in some outcomes may have led to perceived repetition in reporting results.

Through a meticulous examination of these elements, we aim to provide actionable insights and recommendations that pave the way for a future where EHR systems are seamlessly integrated into healthcare practices, ultimately elevating the standard of care and operational efficiency.

The implementation of EHR systems in SSA has heralded a new era in healthcare delivery, particularly in the realm of data management. The findings from various studies converge on a singular truth: EHR systems have significantly enhanced the management of patient data by reducing errors, improving accuracy, and ensuring timely access to crucial information. This discussion synthesizes these findings and interprets their broader implications for healthcare practices and outcomes.

The implementation of EHR systems in SSA has the potential to revolutionize healthcare delivery by significantly enhancing data management practices and patient care. The synthesis of findings from various studies highlights both the triumphs and challenges associated with EHR adoption, providing valuable insights into the steps needed to optimize these systems in our context.

#### Data management

The digitization of patient records through EHR systems has fundamentally transformed data management practices. Traditionally, healthcare facilities relied heavily on manual record-keeping, prone to errors, inconsistencies, and data loss. The studies reviewed illustrate how EHR systems mitigate these issues by streamlining data entry processes and enhancing the accuracy of patient records.

For instance, the integration of mHealth technologies into clinical settings in SSA [34] has reduced manual entry errors and improved data accuracy. This transformation is critical in emergency settings, as evidenced by the usability evaluation at the Komfo Anokye Teaching Hospital in Ghana [17], where timely and accurate documentation facilitated better decision-making during emergencies. Similarly, in Rwanda [15], EHR systems have enabled accurate tracking of patient histories, treatments, and outcomes, significantly improving the management of chronic conditions like Type 1 Diabetes.

#### Enhanced patient care

Improved data management directly translates into enhanced patient care. Accurate and accessible patient records allow healthcare providers to make timely and informed decisions, significantly improving diagnosis and treatment processes. The centralized national EHR system in Gabon [20] demonstrated how coordinated data management across healthcare facilities could facilitate better continuity of care.

#### Addressing challenges

Despite these advancements, significant challenges remain that hinder the full potential of EHR systems. Limited infrastructure, including unreliable power supply and poor internet connectivity, as well as a lack of interoperability with other digital health systems, poses a substantial barrier to EHR adoption. The experiences in Ethiopia [26,39] and Kenya [16,22] underscore the importance of investing in reliable power backup systems and improving internet infrastructure to support continuous EHR operations. We therefore recommend the following to address the identified challenges:

#### Infrastructure investment

Ensuring a reliable power supply and internet connectivity is critical. Investments in backup power solutions like uninterruptible power supplies (UPS) and generators are essential to mitigate the impact of power interruptions on EHR functionality. *Enhance Internet Connectivity*: Collaboration with local governments and Internet service providers is essential to improve Internet connectivity in healthcare facilities, particularly in rural and underserved areas. Implementing offline-capable EHR systems can ensure continuity of care during connectivity issues.

#### Financial support and incentives

Identifying cost-effective solutions, such as open-source EHR systems, and seeking financial support from governmental and international organizations can help mitigate these financial barriers.

#### Robust data security measures

Ensuring robust data security measures, including encryption and secure access controls, is crucial for protecting patient information and building trust in EHR systems.

#### Training and capacity building

Developing comprehensive and ongoing training programmes tailored to the specific needs of healthcare providers can improve proficiency in using EHR systems and enhance user adoption. Continuous support and refresher courses are necessary to keep healthcare providers updated on the latest system features and functionalities.

#### Simplify system interfaces

Ensuring that EHR systems are designed with user-friendly interfaces that minimize complexity and make it easier for healthcare providers to navigate and use the system can improve user acceptance.

#### Technological innovation

Encourage the development and adoption of innovative EHR solutions that address connectivity challenges. Ensuring EHR interoperability with other health information systems is also crucial.

#### Continuous stakeholder engagement

Involving healthcare providers, patients, and policymakers in the planning and implementation process of EHR systems can foster collaboration and ensure their buy-in and support. These recommendations provide a roadmap for addressing the challenges related to EHR adoption and optimizing the implementation of these systems to improve healthcare delivery and patient outcomes. Future research should focus on evaluating the long-term impact of EHR systems on healthcare quality, efficiency, and patient satisfaction in SSA, as well as exploring innovative solutions to overcome existing barriers.

## Conclusion

This scoping review demonstrates that Electronic Health Records (EHR) systems significantly improve data management and patient care in sub-Saharan Africa (SSA). However, to fully realize these benefits, it is crucial to address the challenges related to infrastructure, financial constraints, data privacy, and training. By implementing targeted strategies, SSA countries can enhance the adoption and effective use of EHR systems, leading to better healthcare outcomes and increased patient satisfaction. These findings underscore critical areas of focus for policymakers, healthcare administrators, and technology developers as they strive to integrate EHR systems into healthcare practices effectively. The journey towards seamless EHR adoption is complex, but with strategic interventions, the rewards are substantial in terms of improved healthcare delivery and patient outcomes.

## Supplementary Material

Appendix_2__Data_Abstraction_Instrument_.docx

Appendix_1_EHR_Search_strategy.xlsx

## Data Availability

The data utilized in this review were derived from publicly available sources and literature. As such, all data used in this review are accessible through the respective public and academic repositories, journals, and databases cited within the study. No primary data were collected directly from individuals, and no personal identifiers or confidential information were included.
