# Supplementary material for: A decade of designing and implementing electronic health records in Sub-Saharan Africa: a scoping review
Source: Glob Health Action. 2025 Apr 29;18(1):2492913. doi: 10.1080/16549716.2025.2492913 (PMC12042231; doi:10.1080/16549716.2025.2492913)
Supplement: Appendix_2__Data_Abstraction_Instrument_.docx [file ZGHA_A_2492913_SM0520.docx]

| **Author(s)** | | **Year** | **Study design** | **Study Population** | **Setting** | **Sampling** | **Country** | **Intervention (EHR models, Conceptual framework)** | **Comparator and Outcomes (benefits, challenges)** | **Conclusions/Recommendations** | | |
| --- | --- | --- | --- | --- | --- | --- | --- | --- | --- | --- | --- | --- |
| (1) | | 2021 | Qualitative | Male and female health care workers from two tertiary hospitals in South Africa and Nigeria | The study explored opportunities for integrating mHealth ICTs into the work activities of health care professionals at points of care in clinical settings | Purposive and snowball sampling of 19 (Physicians, nurses, and hospital managers) | South Africa and Nigeria | The ActAD model is a theoretical approach used to describe the elements of work activity and to gain insights into how their interactions inform the development of information systems.  -EHR model: One hospital using smartphones and health ICTs, including a mobile app called VULA, created by Dr William Mapham  One hospital uses a paper-based system  -Both tertiary hospitals used WhatsApp as a means of facilitating communication and sharing patient information. | -Hospital information systems facilitate easier access to and retrieval of patient records electronically during patient visits.  -The VULA mobile app for referrals simplified the referral process and reduced unnecessary referrals to the hospital.  -The voice-to-text feature on WhatsApp assisted healthcare professionals in digitally capturing patient history.  - Health care professionals tend to communicate easily through WhatsApp by sharing patient information, including pictures,  and seeking advice. However, the use of WhatsApp poses potential risks to patient confidentiality  because there are no built-in security measures and consent is a requirement for the exchange of patient information.. | -mHealth ICTs could be used during clinic consultations where physicians need to capture verbal communication with patients and observe their body language.  -The VULA mobile app is useful for managing referrals and information exchange remotely on time, except for its interruptions when physicians are busy with face-to-face consultations and other urgent activities.  -Owing to the mobile nature of nurses, mHealth ICTs could ease information capture, retrieval, and report writing between different points of care, particularly where computer access is limited, and reduce the amount of paper used for patient care administration in hospital settings.  -WhatsApp enables easier communication through instant messaging and offers a means to collaborate between professionals; however, in healthcare contexts, it does not guarantee the privacy of patient’s health information.  -This study encourages ICT and healthcare professionals to work in a transdisciplinary team during the design phase of health technologies. This saves time and costs involved in facilitating training for healthcare professionals in the pilot and post-implementation phases. | | |
| (2) | | 2024 | Quantitative cross-sectional technique | Male and female healthcare personnel involved in the utilisation of the EHR system | Teaching Hospitals | 234 service providers, 9 purposively selected | Ghana | EHR model : EHR System  Lightwave Health Information Management System (LHIMS). | - (LHIMS).was beneficial to Health professionals  and their patients, because it improves work efficiency and workflow of care delivery, and provides the desired output.  -Health professionals perceived positively the implemented EHRs to have several benefits, including improved productivity, enhanced quality  care, easy-to-search patient information, and helped finish work considerably faster.  -Users noted important benefits such as reduced data loss, increased speed and convenience, EHR reducing patient waiting time, and error reduction. -Challenges to the use of electronic records, respondents cited were power fluctuations, lack of training, lack of equipment and frequent breakdown of machines, concerns with privacy and cost of care | -The system's comfort and reliability coupled with the quality of the system providing care and therefore enhancing revenue generation, motivated providers to use the system in delivering services  -The use of the platform had significant challenges. The respondents indicated that, major challenges such as power outages and fluctuations, privacy concerns, frequent breakdowns and inadequate training.  -Recommendation: Future studies should delve into the long-term impacts of provider acceptance and utilisation of the electronic health record system. Exploring how these factors influence patient outcomes, healthcare service quality, and financial sustainability can provide valuable insights. | |  |
| (3) | | 2024 | Cross-sectional with a qualitative component | Health professionals FROM 3 Hospitals in Ghana regarding | Three hospitals in Ghana | 15 participants were purposively selected for the qualitative study, and 234 participants for the  Quantitative | Ghana | Characteristics: their patients’ management component comprises functions used for performing activities such as patient admissions and discharges; searching and retrieval of information; documentation of personal information, medical history, etc.  The clinical component is used for performing functions such as clinical documentation, consultations, medical diagnosis, prescriptions, and provides clinical decision support etc.  The security component has features to enhance the protection of patient data, enforce privacy and confidentiality, and enforce access control. Furthermore, the billing component is used for billing, coding, and accounting. | -Health professionals perceived the system as beneficial to them and their patients, because it improves work efficiency and workflow of care delivery, and provides the desired output.  Health professionals perceived positively the implemented EHRs to have several benefits, including improved productivity, enhanced quality  care, easy-to-search patient information, and helped finish work considerably faster  - Challenges faced by health professionals in using the system emanate from frequent downtime experienced due to erratic power supply or poor internet infrastructure.  -Health professionals, expressed strong displeasure about the EHRs inability to protect  patient data from being accessed by third parties, Unstable internet connectivity and erratic power supply had negative effects on EHRs  use. | -EHR systems in many LMICs continue to improve patients’ safety and increase productivity among health professionals.  -Health professionals have a positive perception of the implemented EHRs, are highly satisfied with them, and are interested in continuing to use them.  -Factors impeding the successful adoption and continuous use of the system, the frequent downtime of the system due to erratic power supply and unstable internet connectivity. Getting a solution around this challenge would mitigate the health professionals’ frustrations and boost their confidence in using the system. Patients will also benefit from a smooth running of the EHRs system.  -Medical practice is increasingly becoming information-intensive, and physicians and other clinicians need to be able to share patient information for decision-making. Therefore, the EHRs system should be able to enforce the privacy, confidentiality, and security of patient information through robust access control. These will build trust among the health professionals and encourage use. | |  |
| (4) | 2024 | Qualitative Study | Health care professional involved in Type1 diabetes individuals management | One clinic and 2 district hospitals purposively selected | 15 purposively selected (nurses and endocrinologists)  Project managers  IT expert/software developer and  Policymakers | Rwanda | Framework: Medical Research Council Framework for developing and evaluating complex interventions.  Theory of change (ToC) model, A ToC model articulates how an intervention is expected to generate outcomes thus,  how the EMR system is expected to link to the expected outcomes.  -“Features” encompasses all the  functionalities of the system and ---“content” encompasses all the  information and data elements of importance to be included in  the EMR system to help monitor and care for T1 diabetes  individuals. | - functionalities integrated into the EMR systems as SMS reminders for consultation, reminders for complications screening, management prompts related to critical clinical values, and missed appointments are important. The system should be able to remind the HCPs when it is time for which tests, ensure timely examinations and avoid repeating exams that are not necessary. The system could also alert about critical test results to help the nurses optimise care  - Strong clinical competence and technical skills are needed to navigate the EMR system  - Offline functionality was viewed as an important  feature related to data protection and ensuring that data are securely captured and uploaded to a backup server. The system can only be sustainable if the system works offline- offline functioning ensures that no data is lost  - Lack of comfort among HCPs to use an EMR system has also been identified as a potential barrier. | -The study concluded that themes related to “features” and“content” are important to identify and consider when developing an EMR system for T1 diabetes management in Rwanda.  -The suggested EMR system is expected to improve data quality, optimize workflows, save more time for patients, improve clinical values, and ensure more patients are referred to treatment for complications on time. Hopefully, this will lead to a slower progression of chronic complications, more research in the area to further improve and optimize care, and eventually a long-term impact on improving health, quality of life, and reduced mortality rates among individuals with T1diabetes. | | |  |
| (5) | 2023 | | Cross-sectional research design | Healthcare workers from a state hospital involved in EHR utilisation | An annexe of the state teaching hospital | 30 purposively selected participants | Nigeria | Framework: Technology acceptance model (TAM)  The ﬁrst stage involves  assessment of the organizational system already in use. The technological aspects are then considered, which are the probable tools and equipment used. The next step includes assessing the  environmental buildings and the personal aspects, including an evaluation of their knowledge  of the use of EHRs, which in this case is computer skills. | -people who were not efficient in the use of Microsoft Excel, word, email usage and WhatsApp functions might have challenges in implementing an EHR system for the management of patients This highlights the need for prioritizing computer literacy as part of the requisite for employment and also the need to train and retrain those already in the system for its use.  -Adopting EHRs will largely depend on the computer skills of the end users before it can be optimally put into use. | -There is a need for continuous training and retraining of existing staff, and computer literacy must be emphasized as part of the requirements for future employment. This will ensure the smooth running of EHRs in hospitals.  -Computer appreciation and knowledge are great determinants for the adoption of EHRs, and if it is going to be introduced into practice there is a need for all staff, no matter their job title, to become acquainted with it. | | |
| (6) | | 2022 | cross-sectional, descriptive and analytical study | Caregivers and administrative staff | Eleven hospitals whose health records were computerized with Open Clinic GA software were included in the study. Five of them belonged to the third reference level (district), two to the second reference level (region), and four to the first reference level (national). Four hospitals were computerized in 2015 and seven computerized in 2017. | Simple random selection of 155 Medical practitioners and specialists, Nurses and Others | Burundi | EHR model: Open Clinic GA. It is an open-source software and can be redistributed or modified, marketable versions are also available. The main modules of the Open Clinic GA software concern the patient's administrative record, the patient's financial record, the patient's medical record, health insurance, credit unions, pharmacy (including stock), laboratory, radiology, health statistics in the form of periodic reports, human resources, the clinical thesaurus with coding assistance validated for ICD-10, SNOMED and multimedia media (images, video, audio) | -the perceived benefits of computerizing medical records are good management of health records and information, continuity and quality of care, good management of resources, reduced service time, ease of the software in producing reports and statistics and easy exchange of information between providers  -The problems expressed by those who were not satisfied are (i) the software malfunction (lack of some diagnostics, updating of information on the EHR, interoperability and report generation problems in some services) the need for training, the insecurity of computers and tablets, network problems, the low capacity of providers to use the tool computer (10.2%), electrical power outages and Difficulties in completing the EHR on time in emergency departments | -This study showed that 44.5% of providers were dissatisfied with the hospital information system based on EHR. Some of the reasons for the lack of satisfaction are lack of updating of information on the EHR, problems of interoperability and generation of reports in some services, the need for training, insufficient computers, problems with networks; the low capacity of providers in the use of the computer tool and power cuts.  -The continuous evaluation of the computerization process and the observations given by users should guide stakeholders to undertake corrective actions to improve the adherence and satisfaction of providers to the hospital information system based on the EHR | | |
| (7) | | 2022 | Cross-sectional, design within an RCT | Health staff from 300 selected health facilities. | 300 multiple level health facilities | 90 users-randomly selected nurses  physicians, social workers, data managers, IT staff, data entry staff | Rwanda | EHR Model: The enhanced OpenMRS electronic health record system. (version 1.11)  OpenMRS has an unusual modular architecture allowing modules from the core development team to be mixed with modules from other developers to create flexible and updatable systems, with typical implementations using 35 to 45 modules. This ensures the core OpenMRS code is common to nearly all OpenMRS installations. Data are stored using a concept dictionary allowing flexibility in data capture and translation to other languages. | - Users strongly agreed EHR makes it easier to manage patients’ medical files and patient’s medical follow-up, makes it easier to make informed decisions, improves the quality of information, and makes it easier to exchange patient information with other health care providers,” provides useful alerts and reminders, enables getting client data easily and/or quickly, helps to generate reliable reports in a short time, stores client information safely and/or securely, and helps to monitor clients daily.  The study showed high levels of dissatisfaction with the EHR and low use levels owing to poor service quality (power infrastructure, user support, training, and lack of computers in  the wards) and the need for double entry of data into the EHR and paper records | -This survey provides evidence that EHR systems have become an accepted component of HIV care delivery  Staff were generally supportive of the system, although most wanted further training, technical support, and better power and network infrastructure.  -Staff at intervention sites were more likely to use or have positive experiences of key functionality that was improved in the enhanced EHR. | | |
| (8) | | 2022 | ToC approach throughout the life cycle of the  implementation | Festac PHC employed 36  health care professionals (HCPs), who served an estimated  population of 27,273 residents | The study was conducted at the Festac PHC in Lagos, Nigeria,  which has the highest number of physicians (7) and a wider  range of health personnel than any other public PHC in Lagos  State | 14 purposively selected participants: physicians, midwives and nurses; and health records  officers | Nigeria | -EHR Model: Open Medical Records System  (OpenMRS) is an EHR software program built for low-resource settings to improve healthcare delivery with the help of a global community that supports the software  -OpenMRS is an open-source program and therefore freely available | -Inconsistencies in EHR data entry during patient encounters occur because of several factors, including human, organizational, and system factors. The willingness of clinical staff to use the new system was lacking because of the perception that the system would add to their existing workload.  - System downtime happens occasionally; when this happens, there is no health IT support technician on the ground to resolve the issue, and hence, the PHC relies on the implementer. | -For new implementers, knowing how to structure this implementation process could be very useful.  -Future health IT implementation in primary care can adapt the ToC approach to their contexts with necessary modifications based on inherent characteristics. | | |
| (9) | | 2021 | Cross-sectional | -The study reported on the development and scaled implementation of mUzima, a mHealth  - Evidence Reporting  assessment (mERA)” checklist was employed to report on the mUzima application. | The “Guidelines for reporting of health interventions using  Mobile phones: mobile (mHealth) evidence reporting assessment  (mERA)” The checklist was developed by the WHO mHealth  Technical  Evidence Review Group to improve  Comprehensivenes and standardization of reporting of mHealth interventions | Not specified, the application was not administered to a sample | LMIC  Kenya, Rwanda, Uganda and Mozambique. | EHR Model: mUzima is an open-source, highly configurable Android application with robust features including offline management, deduplication, relationship management, security, cohort management, and error resolution, among many others. mUzima allows  providers with lower-end Android smartphones (version 4.4 and above) who work remotely to access historical patient data, collect new data, view media, leverage decision support, conduct store-and-forward teleconsultation, and geolocate clients. | -mUzima is an open-source, highly configurable Android application with robust features including offline management, deduplication, relationship management, security, cohort management, and error resolution, among many others.  -mUzima allows providers with lower-end Android smartphones (version 4.4 and above) who work remotely to access historical patient data, collect new data, view media, leverage decision support, conduct store-and-forward teleconsultation, and geolocate clients. | -Greater emphasis needs to be placed on mHealth applications that extend the reach of EHR systems within resource-limited settings to reduce the digital divide that has emerged with the use of standalone EHR systems or mHealth applications.  -mUzima demonstrates how this can be done at scale, with evident adoption across countries and for various types of care programs | | |
| (10) | | 2021 | Quantitative cross-sectional study | 525 Participants (General practitioner/speciality,  Health offices,  Nurse/midwife,  Medical laboratory, Pharmacist and others)  in Eastern Ethiopia | The study assessed the magnitude and factors affecting the utilization of EMR among health professionals in eastern Ethiopia | Stratified sampling technique with proportionate allocation | Ethiopia | EHR Model: EMR is a computerized medical information system that collects, stores, and displays patient information. It can include a wide range of information, including sociodemographics, insurance, medications, intake history, allergies, laboratory test results, immunization, hospitalization history, and others, all while maintaining patient privacy and confidentiality, they are a means of creating legible and organized recordings and of accessing clinical information about individual patients. | -After controlling for confounding variables, sex, educational status, age, work experience, knowledge, attitude, and having EMR training were important factors associated with EMR utilization. | -The utilization of EMR was found to be optimal, and age, work experience, knowledge, attitude, and training were associated with the use of EMR in their facility.  -There should be continuous, targeted, and effective EMR training, and refreshment training once EMR is established  -There should be measures to improve the skills and attitudes of healthcare providers towards the benefits of the implementation  of EMR. | | |
| (11) | | 2021 | Qualitative study | 90 Facility heads at intervention health facilities, 98 pregnant women attending the participating facilities, and 43 policy-makers  From 126 Primary health care facilities | The paper reported whether and under what circumstances using digital interventions to extend health services to remote areas of Nigeria improved the standard of MNCH services | 63 purposively selected Frontline health workers (nurses/midwives, laboratory technicians and community health extension workers (CHEWs), | Nigeria | Framework: The Theory of Change (ToC) model for the eHealth project was collaboratively developed with policymakers and implementers to evaluate the linkages between project inputs, processes, outputs, outcomes, and impacts.  2. complement the above ToC model, we drew on the modified technology acceptance model (mTAM)  proposes that two primary factors influence an individual's intention to use a technology: (i) perceived usefulness or the extent to which the technology will enhance job performance, and (ii) perceived ease of use or the extent to which using the technology will be effortless.  EHR Model: VTR Mobile Application: Enabled users to access video, audio, and text-based MNCH materials through the internet.  Data Digitization Application  Was a tablet computer-enabled point-of-care data capture and decision support tool that enabled users to capture patient-level health information and send appropriate data to remote servers through mobile networks. | -Broad environmental factors that  shaped adoption of technology at the policy level was a supportive policy environment and a thriving private-public partnership between a local technology company and State Governments provided both funding and technical expertise for developing and implementing digital interventions –  - six drivers of acceptance and use of digital technology by health workers. The six drivers of acceptance are: (i) perceived ease of use of technology, (ii) perceived usefulness of technology to enhance job performance, (iii) oﬄine access to clinical videos, (iv) access to tablet computers  in the workplace, (v) prior training to increase familiarity with technology, and vi) ongoing technical support to ensure the functionality of digital technology.  - Barriers that inhibited the use and eﬀectiveness of digital technology in Nigeria were:  (i) increasing workload following technology introduction that prevented FHWs from watching all clinical videos on the VTR platform, (ii) poor internet connection, and (iii) poor electricity  supply that prevented access to the digital platform and recharging of tablet devices in rural areas. | - The range of interventions implemented by the eHealth project in Nigeria led to tangible and reported benefits across all stakeholder groups. - This study demonstrates that simultaneous and sustained implementation of multiple digital technologies at scale enabled via SatCom and 3G mobile networks are viable approaches for strengthening multiple health systems building blocks (in this case, human resources, service delivery, information systems, and governance) to achieve the overall goals of the health system that includes improving the health and wellbeing of people of ages. - Our findings extend the evidence base for the effectiveness of digital health technologies and for theoretical underpinnings to guide further technology use to support improvements in MNCH services in low-resource settings. | | |
| (12) | | 2021 | Secondary data analysis of A prospective cluster randomized trial  clinical to | 3595 patients and 2306 were in the control and intervention arms respectively. | The study assessed the effect of an EHR with a Clinical decision support system (CDSS) compared to EHR only | Block randomization to assign the eligible 13 health facilities into two groups—EHR only (n = 6) or EHR plus CDSS (n = 7) | Kenya | EHR Model: EHR with Clinical decision support system (CDSS)  A CDSS often recommend appropriate action to be taken after comparing specific patient parameters to pre-determined values stored in the EHR’s internal database based on guidelines.  The main effect of the intervention is to inform timely tracing of the defaulting patients or those that are LTFU.  -In the EHR-only (usual care) group, the alerts were turned off in the instance of the EHR installed and there were no individual patient-level alerts printed out nor recommendations filed in the patient charts; the clinical staff relied on weekly summary reports which list all patients who missed appointments to make decisions on follow-up. | -Lower proportion of ART patients who were LTFU compared to those without a CDSS.  - The proportion of ART patients that were LTFU at least once and were traced and linked back to treatment was higher in the sites with a CDSS than those with EHR only.  - | -An Alert-based CDSS implemented as part of an EHR can contribute to enhanced quality of HIV treatment through reduction and early documentation of defaulting and LTFU among HIV patients receiving ART so that follow-up to re-engage clients in care can be activated in resource-limited settings in Kenya.  -Future research is needed on how CDSS can best be combined with other interventions to reduce LTFU. | | |
| (13) | | 2021 | **evaluation study** using **computer-generated data** | The study was conducted using census method with all 376 facilities that had  KeEMRs implemented between 2012–2019 eligible to participate. | This study evaluated the actual use ofKeEMRs within the facilities in which  the system is deployed to inform actual EHRs usage across the country, based on computer-  generated data. | All the 376 facilities implemented with KeEMRs were approached to participate in the study.  Nevertheless, data collection script was distributed to 312 sites that gave authority for the com-  mencement ofthe study and had used the EHRs for at least six months. | Kenya | EHR Model :KenyaEMR system (KeEMRs):  KeEMRs is an implementation and adaptation of the open-source OpenMRS system platform, which is widely deployed in many countries in Africa. KeEMRs support both retrospective and point-of-care data entry (RDE & POC) with most of the facilities equipped for POC implementation. | The primary outcome of interest for this study was to determine the collective performance by facilities on each of the seven indicators throughout KeEMRs implementation in Kenya, as a measure of overall EHRs usage.  -Indicators reflecting system use and interoperability domains indicated low measures, suggesting the need for further improvement.  - The study established a slow incorporation of the interoperability layer (IL) within the EHRs. Hence could not exchange health data with an external system | -Assessment of the actual use of implemented EHRs within LMICs is important. The systematically generated standard EHRs usage indicators can be adopted and used successfully within facilities across countries.  -Results from this study demonstrate that there are many areas of improvement in EHRs use, as well as the need for continuous monitoring of EHRs use to inform timely interventions.  -Simply counting the number of implementations, as is currently done in many settings, remains a highly inadequate measure for evaluating EHR implementation success | | |
| (14) | | 2020 | survey-based cross-sectional study. | The target population consisted of professionals in  charge of direct patient care (physicians, nurses, mid-  wives, etc.) or people working in healthcare services  (managers, administrators) employed in different health  structures | All health facilities in the 10 regions of Gabon. | A convenience sample of twenty-six hundred seven (2327) ( Nurse,  Other health professionals, midwives, General practitioners, Administrators,  Specialist practitioner) | Gabon. | Theoretical model  The theoretical background of this study is inspired by the DeLone and McLean Information System Success Model (ISSM). This model recommends taking into account five variables to measure the success of the implementation of an information system: system quality (SQ), support quality (SupQual), information quality (IQ), actual use (AU), satisfaction, and net benefits (Impact). | -Providers’ perceptions of the positive impact resulting from the use of the HIS were explained by five variables of our adapted model, namely Support Quality, Information Quality, System  Quality, Actual Use, and Useful Functions | -Using an adaptation of the Information System Success Model, this study found that information quality, system quality, support quality, actual use, and useful functions influenced the perception of positive impacts of the HIS by healthcare providers.  -Thus, to ensure the success of the implementation of the national HIS in Gabon, it is essential to involve healthcare providers in the design of this system and make sure that it can be incorporated into their clinical practice. Such cooperation is needed to ensure the quality of the system in terms of usability, quality of information, and end-user support.  -Potential users should also be trained in the various functionalities of the HIS so they can see and evaluate its benefits firsthand and increase the likelihood of its successful use. | | |
| (15) | | 2024 | Cross-sectional study | Trained pharmacy providers | 20 private pharmacies in Kisumu, Kenya | 1,691 Pharmacy clients (≥18 years) recruited through convenience sampling  -40 Pharmacy providers: Purposively selected | Kenya | EHR Model  Artificial intelligence (AI), Audere developed an AI algorithm specifically for interpreting Mylan HIVSTs. | -The AI algorithm did not miss a single HIV infection and, importantly, outperformed humans at correctly identifying positive tests as positive.  - AI algorithm erred, it erred in the more conservative direction, producing some false-positive—but zero false-negative—interpretations. | -AI computer vision technology shows promise as a quality assurance tool for HIV testing. Such technology may be especially useful for enabling HIV services to be delivered outside of traditional healthcare settings, by new cadres of providers, and/or at different cadences to better meet client needs and preferences and to use existing health resources more efficiently.  --Future research should also explore AI biases to minimize biases that may compromise care quality, fairness, and equity. | | |
| (16) | | 2022 | Cross-sectional | 1,177 clinicians (doctors, nurses, laboratory scientists/technologists and Pharmacists) and administrators at the managerial level | This study was conducted in four states in the Niger Delta  region viz: Akwa-Ibom, Cross Rivers, Rivers and Imo states | Stratiﬁed random sampling was used to  Select healthcare facilities that participated in the survey and selection of  respondents from each healthcare facility  1,177 clinicians purposively selected(doctors, nurses, laboratory scientists/technologists and Pharmacists) and administrators at the managerial level | Nigeria | -Framework: Technology Acceptance Model (TAM) to the prediction and explanation of end-user reactions to health IT. TAM is based on the theory of reasoned action (TRA) and hypothesizes that Perceived Usefulness and Perceived Ease of Use, are of primary relevance for technology acceptance  -4 major factors underpin acceptance of health technology: performance expectancy, effort expectancy, social influence, and facilitating conditions | -The majority of respondents had an intermediate level of computer literacy which can directly impact their willingness to adopt and use systems  -usefulness of new technology was an important facilitator to willingness to adopt and use the technology  -Perceived ease of use and awareness which are important factors for adoption in this study have been linked with training, knowing the beneﬁts of EMR, access to information, and improved knowledge and experience of EMR.  -lack of constant supply of electricity as well as  poor internet services are barriers to the successful adoption and implementation of a nationwide EMR  -user perception of risk and safety of their data when using EMR decreases their propensity to adopt EMR. | -Infrastructural availability such as lack of or poor internet connectivity and lack of constant supply of electricity were not seen as barriers to the successful adoption and implementation of an EMR system. While this may be due to dependence by many  hospitals in Nigeria on alternative power supply for their operations, however, this remains a huge challenge, especially  in peripheral facilities as EMR may not be used consistently because of the constant power outage. | |  |
| (17) | | 2021 | Cross-sectional | -The study described in detail each element of the evidence reporting assessment (mERA)” checklist developed by the WHO mHealth Technical Evidence Review group to  Improve comprehensiveness and standardization of reporting of mHealth interventions. | Inter-country piloting of mUzima is an open-source, highly configurable Android application | 4 countries (Kenya, Rwanda, Uganda and Mozambique) | Kenya, Rwanda, Uganda and Mozambique. | -mUzima is an open-source, highly configurable Android application with robust features including offline management, deduplication, relationship management, security, cohort management, and error resolution, among many others. mUzima allows providers with lower-end Android smartphones (version 4.4 and above) who work remotely to access historical patient data, collect new data, view media, leverage decision support, conduct store-and-forward teleconsultation, and geolocate clients | -mUzima is a robust and adaptable Android-based mHealth platform that can seamlessly interoperate with the OpenMRS EHR system  -mUzima was developed as an open-source application under the Mozilla Public License 2.0 license The application has a modular architecture that lends it the strength of simplicity while ensuring full functionalities of the installed modules  -Access to the mUzima mobile application requires a username and password, which are initially authenticated against the EHR system.  -mUzima serves as a HIS for collecting primary health data, which are then exchanged with the associated EHR system. Health information exchange between mUzima and OpenMRS  is achieved through the use of the same concept dictionary terms, locations, providers, and patient identifiers that are common between the 2 systems. | -Although EHR and mHealth solutions have been widely embraced in LMICs, there is often a lack of seamless data exchange between widely deployed EHR systems and these mHealth solutions.  -greater emphasis needs to be placed on mHealth applications that extend the reach of EHR systems within resource-limited settings to reduce the digital divide that has emerged with the use of standalone EHR systems or mHealth applications. mUzima demonstrates how this can be done at scale, with evident adoption across countries and for various types of care programs | |  |
| (18) | | 2020 | Cross-sectional | All 414 health professionals were included in the study | The study determined health professionals' readiness and associated factors toward the implementation of EMRs in four selected primary hospital | All 414 health professionals were included in the study | Ethiopia | - A self-administered questionnaire and in-depth interviews to interview the key informants | -Health professionals who had good knowledge of the EMR system were about 2.64 times more likely to be ready for an EMR system as compared to health professionals with poor knowledge  -Health professionals who had a favourable attitude toward an EMR system were 2.63 times more likely to be ready than their counterparts.  Study participants who had their personal computers were about 2.34 times more ready for an EMR system as compared to those study participants who had no private or personal computers.  -Study participants who were computer-literate were 3.3 times more ready for an EMR system than their counterparts  -Health professionals who had taken EMR training were about 3.63 times more ready for an EMR system as compared to those health professionals who had not taken any | -EMR knowledge, attitude toward an EMR system, having personal computer literacy, and EMR training were the significant factors for EMR readiness.  -Availing ICT infrastructure and provision of training for health professionals is crucial for the adoption of EMR systems.  -Promotion of EMR systems by the health program managers to the potential users is also important to increase the adoption and success rate of EMR systems | |  |
| (19) | | 2022 | Qualitative | Health professionals including doctors and nurses in clinical departments that use health ICTs for medical imaging, referrals and reporting. | The study identified how unintended consequences disrupt technology-enabled work activities of healthcare professionals in hospital settings. | A total of 19 participants purposive sampling technique from two tertiary hospitals | South Africa | -The technique of enquiry was semi-structured interviews to enable non-binary answers and further probing  -Each of the interviews lasted 35–40 min, and was recorded with a voice recorder to capture all information  -suitability was perceived as the extent to which health ICT fits the purpose of a work activity. This is attributed to the awareness and experiences of healthcare professionals’ pre-implementation and technical know-how to utilize health ICTs in the intended context of use. | -The suitability of health ICTs for the work activities of healthcare professionals is closely tied to its usefulness. The usefulness of health ICTs is largely dependent on the extent of their usability as experienced by information  -It is evident from the findings that there is a need for an in-depth understanding of the impact and role played by the context of use when investigating unintended consequences associated with health ICTs  Doctors expressed a feeling of satisfaction attributed to how health ICTs simplified access to patient records to enable decision-making.  -Healthcare professionals experience delays due to the number of interruptions and downtime caused by health ICTs at points-of-care within healthcare systems overwhelmed by the daily number of patients that seek medical attention | -The study established that when technology-enabled work activities at points of care are in contradiction with the contextual conditions and complexity of tasks performed by healthcare professionals, it results in unintended consequences.  -Unintended consequences experienced by healthcare professionals are: repetition of tasks, unanticipated delays; use of alternate means to retrieve patient records, and disruptive patient consultations.  -The current versions of hospital information systems are fit for purpose but cause disruptions and delays to the work activities of healthcare professionals despite their benefits to facilitate remote communication, enable information sharing and quicker access to patient records.  -Understanding unintended consequences associated with technology-enabled work activities at points of care could assist the industry vendors in improving how health ICTs are designed to improve the work experiences of healthcare professionals at points of care in hospital settings | |  |
| (20) | | 2021 | Cross-sectional | All health professionals permanently working in Illu Aba  Bora and Buno Bedele zones, southwest Ethiopia were  eligible in this study | The  study was conducted at public hospitals in Illu Aba Bora  and Buno Bedele Zones, Oromia Region, Southwest Ethi-  opia. The capital city of Illu Aba Bora and Buno Bedele  is Mettu and Bedele respectively. Mettu and Bedele cities  are located 600 km and 580 km away from Addis Ababa,  the capital city of Ethiopia. | A total of 423 healthcare providers working in public hospitals were selected using a simple random sampling technique | Ethiopia | -This study assessed healthcare providers’ EHR readiness and associated factors in Southwest Ethiopia. | -Readiness to adopt EHRs was interlinked with socio-demographic, behavioural, technical, technological, and organizational factors. Healthcare providers aged below 30 were more likely to be ready to adopt EHR compared to those aged above 30.  -The odd of healthcare providers who had favourable attitudes were more likely to be ready than those who had unfavourable attitudes.  -Healthcare providers who had good EHR awareness were more likely to be ready  -Computer literacy played an important role in determining healthcare providers’ readiness to adopt EHRs in which respondents with high computer literacy were more ready than their counterparts  -The study revealed healthcare providers were more likely to be ready when they believed that EHR is more beneficial.  -Healthcare providers who got computer access at  facilities were ready for EHRs. | -Around half of the respondents had a good level of overall readiness for the adoption of EHR which was considered inadequate. This finding implied that a huge effort is required to improve readiness before the actual implementation of EHRs  -Enhancing computer literacy, building their confidence to raise self-efficacy with such technology, building a positive attitude, awareness campaign of HER, and recognizing the usefulness of such systems were the necessary measures to improve EHR readiness in this setting.  -Recommendation: Further studies are recommended to encompass all types of EHR readiness such as organizational readiness, technology readiness, societal readiness, and so on.  -Additionally, exploring healthcare providers’ opinions with qualitative study and extending the proposed study to other implementation settings are recommended to be addressed by future works | |  |
| (21) | | 2019 | Qualitative retrospective | All health service providers involved in the use of SmartCare, a Zambian Ministry of Health (MoH)-led  project funded by the US Centres for Disease Control  and Prevention (CDC) | The SmartCare database is a derivative of the PTS  (patient tracking system), which was developed in 2004,  based on a health facility-centred EHR system. In 2010,  It became a national health programme and  was then rolled out throughout the country. It is simple-  supported by the government, and both international and local  organisations primarily for patient management | Data were generated from 17 in-depth semi-structured  face-to-face interviews, 4 data entry observations, and 3 FGDs with 22 pregnant and lactating women seeking  PMTCT services from 3 health facilities | Zambia | - SmartCare was developed to improve continuity of care and provide timely data on maternal and child health, HIV/AIDS, tuberculosis and malaria interventions for public health purposes, trend reporting and analysis for health officials and clinicians | -The completeness of paper-based records was slightly better than electronic records.  -The workload affected the documentation, as evidenced by poor data quality in the quantitative analysis of SmartCare PMTCT data  - There was a notable lack of appreciation of the system, and a need to train and support end users of the system such as the clinicians who are directly involved in the data collection process. | - The SmartCare system has structural challenges which can be traced to its development. Funding gaps have resulted in staffing and data collection disparities within  IPs. The lack of feedback from the system has also led to complacency at the operational level, which has resulted in poor data quality in later years. The data from the database, if appropriately understood, could be used by  health facility staff as an advocacy tool, as well as in monitoring the impact of the PMTCT programme. Our research could aid other countries wanting to develop their own EHR systems. | |  |
| (22) | | 2023 | Cross-sectional | Healthcare professionals working at OHC Ado Ekiti, which comprises of 4 nurses,  13 community health extension workers, 5 attendants, 1 health assistant, 1 pharmacy techni-  cian, 1 laboratory technician, 3 doctors, and 2 health information management technicians | Okela Health Centre (OHC), an annex of Ekiti State  University Teaching Hospital Ado-Ekiti, in southwest Nigeria.  The study aimed to  determine the computer skills required for the uptake of electronic health records (EHR) by healthcare workers in an annexe of the state teaching hospital | No sampling, the study included all health care workers at OHC Ado Ekiti totaling to 30 respondents | Nigeria | -Adopting EHRs, as with other technologies, may be determined and explained by the use of the technology acceptance model (TAM).  - Adopting EHRs, as with other technologies, may be determined and explained by the use of the technology acceptance model (TAM)  -Using the TAM diagram, when a technology is introduced, the first stage involves assessment of the organizational system already in use | -The technology organization environment (TOE) framework, where computer processing power, computer experience, computer knowledge, perceived usefulness of intelligent systems, and their ease of use are seen as factors influencing the adoption of intelligent healthcare services by medical institutions with integrated medical care | -There is a need for continuous training and retraining of existing staff, and computer literacy must be emphasized as part of the exquisite for future employment. This will ensure the smooth running of EHRs in hospitals. Computer appreciation and knowledge are great determinants for the adoption of EHRs, and if it is going to be introduced into practice there is a need for all staff, no matter their job title, to become acquainted with it. | |  |
| (23) | | 2014 | Before and after the design | ll patients aged 2 years and older enrolled at selected clinics  This excluded 2414 patients who had transferred in from other  clinics. | Nyanza Province with highest HIV burden among Kenya’s  eight provinces, (14.9%); it is home to about a  third of all HIV-infected persons in Kenya.12 EMRs were installed at  17 health facilities that had electricity and adequate security for  computers. The 17 clinics, which were providing HIV care and  treatment to about 39 203 active patients as of September 2012 | A total of 37 851 patients aged 2 years and older were enrolled at the clinics, and 17 health facilities providing HIV care and treatment services in Nyanza Province, western Kenya. | Kenya | -A retrospective study was carried out to assess the quality of pre-ART care using three indicators: (1) the performance of a baseline CD4 test, (2) the time from enrolment in care to the first CD4 test, and (3) the time from baseline CD4 to second CD4 test. A comparison of these indicators was made pre- and post-the introduction of an EMR system in 17 rural HIV clinics. | -EMR use was associated with better adherence to the pre-ART care guidelines in all three of the outcome measures  -The EMR was associated with a 59% increase in the odds of performing a baseline CD4 test.  -There was a significant reduction in time from enrolment into pre-ART care to the first CD4 test. EMR use was associated with a 47% higher hazard of conducting a baseline CD4 test.  -, we found data quality to be better in the EMR compared to the paper system. For example, key data elements such as the date of the CD4 test and CD4 results were three times more likely to be missing in the paper system compared to the EMR. This could be attributed to the fact that the EMR contains mandatory fields that must be entered, resulting in fewer missing values | -EMR seems to have improved the quality of patient care.  -Further work on the evaluation of the use of clinical decision support functionality of an EMR on compliance with pre-ART guidelines is needed to provide a more complete picture of the effect of EMRs.  -The study demonstrated the use of the EMR to be positively associated with enhanced compliance with key quality indicators for pre-ART care, as required by the HIV treatment guidelines. EMRs have a potential positive impact on the quality of care for HIV patients in the resource-constrained setting. However, there is still the need for much greater improvement. | |  |
| (24) | | 2020 | Cross-sectional | -This paper presents a design model that can be implemented for EHR rollout in Sub-Saharan Africa | This paper presents a design model that can be implemented in these economies. A workflow of operations in a typical hospital | All the caregivers’ work-  stations are connected to the EHRs via a conﬁgured server | Nigeria | -The database was designed around the typical workflow of a paper-based health institution, as shown  -If standards for interoperability are available, these can be accommodated in the design  -The patients’ database table holds all health information about the patients from bio-data to health history.  -The officials' table was created to hold information about healthcare personnel to control access to the EHR. | -A workflow of operations in a typical hospital was defined and used in the design process.  -The patients’ data are captured through the front-end application, and these are stored in the database.  -A role-based access level is assigned to the caregiver through the incorporated fingerprint access control.  -All the caregivers’ work stations are connected to the EHRs via a configured server. The designed EHR system will enable optimization of clinical practices and management functions when deployed. | -The system is affordable to clinics and hospitals in the developing world.  -It can be adapted to meet the specific goals or requirements of an organization  -The built web-based application is capable of the addition or removal of patients and healthcare providers, as it is necessary  -The EHR systems have a flexible design and good interoperability between different parts of the system.  -Recommendations:  -there is a need to periodically upgrade the functionality of the EHRs for sustainability. -The functionality of the EHR system can be enhanced through the inclusion of additional components, such as a research facility and medical entities dictionary. Also, adequate security measures are required to prevent a breach of privacy and EHR system collapse. | |  |
| (25) | | 2018 | An institution-based comparative study, supplemented with a qualitative approach | A total of 4,907 ART clients on follow up at the ART clinic of the hospital. | The study was conducted at the University of Gondar Referral Hospital, the oldest and biggest  referral hospitals serving for more than five million people in North West Ethiopia. The hospital  has many specialized clinics, including for chronic illnesses (cardiac disease, renal disease and  diabetes), mental health and HIV care. | 250 patient records were found to have both electronic and paper-based records. All these 250 patient records were included in the study. | Ethiopia | -Both paediatric and adult ART records were used.  -All patients on ART for at least six months, having complete records both paper-based and electronic (in the local EMR system called SmartCare) and having a follow-up visit in the ART clinic, were included in the study.  -There was a total of 4,907 ART clients on follow-up at the ART clinic of the hospital. Of these, 250 patient records were found to have both electronic and paper-based records.  -completeness and reliability of the records, checklists were developed based on the national ART data elements  -Qualitative data was obtained by in-depth interviews of key informants that included physicians, nurses, health officers, data clerks and ART case managers | -The study showed the overall completeness of medical records in the paper-based version was still slightly higher than the EMR.  Varying data completeness was observed among the specific data elements. For instance, the completeness of patient weight data and pregnancy status was higher in paper-based than in EMR. Data elements such as WHO staging, functional status, TB screen, next visit date, opportunistic infections (OIs)screening and prophylaxis for OIs have also significantly better levels of completeness in paper-based records than in EMR.  -In both paper-based medical records and EMR, the completeness of HIV care and ART data elements was generally lower than the completeness of sociodemographic data elements  The major evidence in this research is the impact of dual documentation on the quality of the data: taking the time of health workers. | -The overall ART data quality was still slightly better in paper-based records than in the electronic medical record system.  -The main reason affecting the EMR data quality was the current dual documentation practice both on paper and electronic, for each patient in the hospital.  -Recommendation: The hospital management needs to decide to use either the paper or the electronic system so that health workers can save time by a single documentation practice.  -Training and continuous support to health workers is recommended to build the capacity of health workers on data documentation practices | |  |
| (26) | | 2020 | Cross-sectional | -Publications on EHR were reviewed for the role of EHR in insurance schemes | PubMed and Embase databases, as well as  Google Scholar. We restricted our search to publications  in English | 76 publications were extracted. | Nigeria | -Data extraction was conducted using an interpretive approach by the two authors as independent reviewers based on an identical data extraction form to synthesise the different studies, and we used all the  -We also focused on how suitably robust EMR systems could be used to realistically mitigate some of the challenges (acts of forgery, counterfeiting and other forms of fraud) | -Evidence suggests that the current schemes in the subregion are unsustainable and face extremely high risks of failure because of the difficulty in preventing the problems of fraud and other deliberate acts of abuse under their current paper-based operations  -Indeed, the EMR system has become a tool of necessity for healthcare delivery in developed countries – facilitating access to standard operating procedures; treatment guidelines and the provision of comprehensive, updated information on prescription drugs, for the minimisation of contraindications, drug-drug interactions and monitoring the health status of patients | -The review highlights the gap in the literature on the peculiar challenges associated with public health insurance schemes in sub-Saharan Africa and extends our knowledge by describing some of the fundamental problems – issues which are largely ignored, despite the proliferation of these schemes in recent years.  -It describes how some of those associated with the environment of poor infrastructures, inadequate management and highly fraudulent activities can be addressed with suitably designed EMR systems, suggesting that such resolutions may enhance the sustainability of these schemes, which are vital to the goal of universal health coverage. | |  |
| (27) | | 2014 | Retrospective, pre-post EMR study | The study population consisted of male and female patients  aged 2 years or older, enrolled in HIV care not more than  1 year prior to the implementation of an EMR at the clinic. For example, we excluded patients enrolled in HIV care before June 2008 at clinics where an EMR was installed in June 2009. | The study assessed the effect of transmission from paper-based (Comprehensive Care Clinic Card) to the EMR system (Comprehensive Care  Centre Patient Application Database (C-PAD) | This study was conducted at 17 out of 122 rural health facilities in Siaya County in Western Kenya, providing HIV care and treatment services. The 17 government-owned clinics are among 20  facilities where the Kenya Medical Research Institute (KEMRI)  provides data management and  Information and communication technology (ICT) support.  7298 records for patients  aged 2 years or older, enrolled in HIV care | Kenya | The outcomes analysed were: (1) the proportion of patients eligible for  ART based on CD4 count or WHO clinical staging who initiate therapy; (2) time from eligibility for ART to actual ART; and (3)  time from ART initiation to the ﬁrst CD4 T-cell count test following ART.  -Outcomes were compared before (paper-based system) and after (C-PAD EMR) the EMR system was introduced in the facilities | -EMR use was associated with a 22% increase in the odds of ART initiation among eligible patients enrolled on HIV care after transitioning from a paper-based system  -The study also showed that about 80% of patients eligible for ART had been initiated on therapy.  -EMR use increased the chances of patients turning up at the clinic for ART initiation, | -EMRs can improve the quality of HIV care through the appropriate placement of ART-eligible patients on treatment in resource-limited settings. However, other non-EMR factors influence the timely initiation of ART.  -Rigorous evaluation studies are needed to demonstrate associations between decision support systems implemented in EMRs and important quality of HIV care indicators such as retention on treatment. | |  |
| (28) | | 2018 | A literature review was conducted | A comprehensive literature search was conducted on two electronic databases: PubMed and Medline. The Google scholar search engine was also utilised as well as organizations’ websites, such as those of the WHO  and ISO | A literature review was conducted using one of the second-generation approaches: narrative synthesis (a mixed-methods approach) | Forty-seven papers were retrieved.  Publications of interest were those published in English and with information on factors that limit the  implementation/adoption of EHR as well as factors/strategies that will improve its adoption in sub-Saharan  Africa. Exclusion criteria were studies that were not published in English as well as those that were not on  developing countries | Sub-Saharan African countries. | -A comprehensive literature search was conducted on two electronic databases: PubMed and Medline.  -After screening, twenty-one papers were included in the final review.  -Inclusion and exclusion criteria were based on matching types of evidence to research purposes based on their relevance and quality of individual studies.  -Dixon-Woods et al.(2006) were adopted for quality assessment, to exclude the fatally flawed papers | -The most frequently reported major factors that limit EHR implementation in sub-Saharan African countries are as follows: high initial and ongoing maintenance costs, lack of financial incentives for adoption, lack of priorities, poor electricity supply, lack of internet connectivity, low computer literacy level, some of these identified factors are similar to findings in the developed part of the world  -Factors Facilitating EHR Implementation in Sub-Saharan Africa: Comprehensive planning before implementation, Training of EHR users, Financial Support as incentives for utilisation, Appropriate EHR System Selection, and Phased Implementation. | -The main drivers for the increasing interest in EHR include the need to improve efficiency in healthcare service delivery, improve patient safety, increase access to healthcare services, and more importantly, the need to reduce the costs of medical expenditures.  - Factors that limit broad adoption include high initial costs of procurement of the EHR system and ongoing maintenance costs, lack of financial incentives for adoption, lack of priorities, poor electricity supply, lack of internet connectivity, primary users’ limited computer skills, and lack of robust healthcare infrastructure. --Improved efforts, such as the inclusion of the biomedical informatics program in medicine, pharmacy, nursing, and other potential users of EHR curricula and the establishment of computer laboratories, are required to increase the students' access to computers and the internet. |  |  |
| (29) | | 2018 | Landscape review of existing literature | -This study describes a novel idea: to develop and deploy an EHR using existing open-source software for use in public health facilities in Kenya.  -Clinicians and IT staff were the participants | This case study has been developed in 2 phases over a period  of 2 years. In the first phase, a research team from Kenya  Medical Research Institute (KEMRI)/Wellcome Trust Research  Programme (ME, JM, NM) supported by the University of  Oxford (CP) was commissioned by the MoH and WHO to report  on the initial plans and progress of the AfyaEHMS project | County referral hospital and health centers | Kenya | -Kenya developed an eHealth strategy and implemented two major health information technology projects: District Health Information Software Version 2, for collating national health care indicators and a rollout of the Kenya  The implementation process included upgrading the hospital information technology infrastructure, training users, and attempting to garner administrative and clinical buy-in for the adoption of the system. | -The lack of power, inadequate hardware, and networking were a major challenge to system setup during the deployment. For this project, the implementing team addressed the power and hardware issues by adding extra local human resources for troubleshooting and fixing issues as they arose  -Use of open-source software may offer some respite from the high costs of proprietary software, which is a well-documented barrier to the adoption of EHRs. | -Implementing EHR systems is a challenging process in high-income settings. In low-income settings, such as Kenya, open-source software may offer some respite from the high costs of software licensing, but the familiar challenges of clinical and administration buy-in, the need to adequately train users, and the need for the provision of ongoing technical support are common across the North-South divide. | |  |
| (30) | | 2016 | Cross-sectional | All the health professionals working in the hospital were the study population of this study. Health  professionals with less than 6 months of working experience or absent from their work due to annual  leave and maternity issues were excluded from being study participants in the study. | Ayder Referral Hospital is located in the Mekelle city administration, the capital city of the Tigray Regional State, one of the regions in the nine administrative regions of Ethiopia. The city is located about 780 km away from Addis Ababa, the capital city of Ethiopia | A total of 428 health professionals participated in the study, with a response rate of 86%. | Uganda | -Stre@mline is an EHR platform that has been locally developed by Ugandan clinicians and engineers in Southwestern Uganda.  -It is tailored to the specific context and the needs of low-resource hospitals.  -It operates without internet access, incorporates locally relevant standards and key patient safety features, has a medication inventory management component, | -The resulting product is a sustainable and scalable EHR system that addresses many of the shortcomings in existing EHR platforms described earlier.  Stre@mline has also improved resource planning by allowing pharmacists to track their drug stocks in real-time | -Stre@mline is a locally developed electronic health record system tailored to the specific needs of resource-constrained settings. It is unique in that it is entirely locally developed through a partnership between a local hospital and a local technology company, and is developed and sustainable without funding from outside Uganda. The  -The embedded guidelines and triage assistance within Stre@mline have also substantially improved patient care, with 100% of respondents agreeing that it has improved their “decision-making” | |  |

**References**

1. Ogundaini OO, De La Harpe R, McLean N. Integration of mHealth information and communication technologies into the clinical settings of hospitals in Sub-Saharan Africa: Qualitative study. JMIR Mhealth Uhealth [Internet]. 2021 Oct 1 [cited 2024 Oct 29];9(10):e26358. Available from: https://mhealth.jmir.org/2021/10/e26358

2. Antor E, Owusu-Marfo J, Kissi J. Usability evaluation of electronic health records at the trauma and emergency directorates at the Komfo Anokye teaching hospital in the Ashanti region of Ghana. BMC Med Inform Decis Mak [Internet]. 2024 Dec 1 [cited 2024 Oct 24];24(1):1–14. Available from: https://pubmed.ncbi.nlm.nih.gov/39169338/

3. Mensah NK, Adzakpah G, Kissi J, Abdulai K, Taylor-Abdulai H, Johnson SB, et al. Health professionals’ perceptions of electronic health records system: a mixed method study in Ghana. BMC Med Inform Decis Mak [Internet]. 2024 Sep 16 [cited 2024 Oct 24];24(1):254. Available from: https://bmcmedinformdecismak.biomedcentral.com/articles/10.1186/s12911-024-02672-3

4. Bille N, Christensen DL, Byberg S, Calopietro M, Gishoma C, Villadsen SF. The Development of an Electronic Medical Record System to Improve Quality of Care for Individuals With Type 1 Diabetes in Rwanda: Qualitative Study. JMIR Diabetes [Internet]. 2024 Sep 20 [cited 2024 Oct 29];9(1):e52271. Available from: http://www.ncbi.nlm.nih.gov/pubmed/39303284

5. Sibiya MN, Akinyemi OR, Oladimeji O. Computer Skills and Electronic Health Records (EHRs) in a State Tertiary Hospital in Southwest Nigeria. Epidemiologia [Internet]. 2023 Jun 1 [cited 2024 Nov 21];4(2):137. Available from: https://pmc.ncbi.nlm.nih.gov/articles/PMC10204356/

6. Munezero F, Sossa CJ, Nyandwi J, Bazira L. Provider satisfaction with the health information system based on the electronic health records in Burundi’s hospitals. Int J Community Med Public Health [Internet]. 2022 Jul 27 [cited 2024 Oct 29];9(8):3111. Available from: https://www.ijcmph.com/index.php/ijcmph/article/view/9954

7. Fraser HSF, Mugisha M, Remera E, Ngenzi JL, Richards J, Santas X, et al. User Perceptions and Use of an Enhanced Electronic Health Record in Rwanda With and Without Clinical Alerts: Cross-sectional Survey. JMIR Med Inform [Internet]. 2022 May 3 [cited 2024 Oct 29];10(5):e32305. Available from: http://www.ncbi.nlm.nih.gov/pubmed/35503526

8. Adedeji T, Fraser H, Scott P. Implementing Electronic Health Records in Primary Care Using the Theory of Change: Nigerian Case Study. JMIR Med Inform [Internet]. 2022 Aug 11 [cited 2024 Oct 29];10(8):e33491. Available from: http://www.ncbi.nlm.nih.gov/pubmed/35969461

9. Were MC SSMBMSRNCPYA. mUzima Mobile Electronic Health Record (EHR) System: Development and Implementation at Scale. J Med Internet Res [Internet]. 2021 [cited 2024 Oct 29]; Available from: https://www.jmir.org/2021/12/e26381/

10. Oumer A, Muhye A, Dagne I, Ishak N, Ale A, Bekele A. Utilization, Determinants, and Prospects of Electronic Medical Records in Ethiopia. Kottayasamy Seenivasagam R, editor. Biomed Res Int [Internet]. 2021 Nov 8 [cited 2024 Oct 29];2021:1–11. Available from: https://www.hindawi.com/journals/bmri/2021/2230618/

11. Ebenso B, Okusanya B, Okunade K, Akeju D, Ajepe A, Akaba GO, et al. What Are the Contextual Enablers and Impacts of Using Digital Technology to Extend Maternal and Child Health Services to Rural Areas? Findings of a Qualitative Study From Nigeria. Front Glob Womens Health [Internet]. 2021 [cited 2024 Oct 29];2. Available from: https://www.frontiersin.org/journals/global-womens-health/articles/10.3389/fgwh.2021.670494/full

12. Oluoch T, Cornet R, Muthusi J, Katana A, Kimanga D, Kwaro D, et al. A clinical decision support system is associated with reduced loss to follow-up among patients receiving HIV treatment in Kenya: a cluster randomized trial. BMC Med Inform Decis Mak [Internet]. 2021 Dec 1 [cited 2024 Oct 29];21(1):1–11. Available from: https://login.research4life.org/tacsgr1bmcmedinformdecismak_biomedcentral_com/articles/10.1186/s12911-021-01718-0

13. Ngugi P, Babic A, Were MC. A multivariate statistical evaluation of actual use of electronic health record systems implementations in Kenya. PLoS One [Internet]. 2021 Sep 1 [cited 2024 Oct 29];16(9):e0256799. Available from: https://journals.plos.org/plosone/article?id=10.1371/journal.pone.0256799

14. Bagayoko CO, Tchuente J, Traoré D, Moukoumbi Lipenguet G, Ondzigue Mbenga R, Koumamba AP, et al. Implementation of a national electronic health information system in Gabon: a survey of healthcare providers’ perceptions. BMC Med Inform Decis Mak [Internet]. 2020 Dec 24 [cited 2024 Oct 29];20(1):202. Available from: https://bmcmedinformdecismak.biomedcentral.com/articles/10.1186/s12911-020-01213-y

15. Roche SD, Ekwunife OI, Mendonca R, Kwach B, Omollo V, Zhang S, et al. Measuring the performance of computer vision artificial intelligence to interpret images of HIV self-testing results. Front Public Health [Internet]. 2024 [cited 2024 Oct 29];12:1334881. Available from: http://www.ncbi.nlm.nih.gov/pubmed/38384878

16. Akwaowo CD, SHM, EN, ICM, ANF, MO, DE, UE, EV, & UF. Adoption of electronic medical records in developing countries—A multi-state study of the Nigerian healthcare system. Front Digit Health [Internet]. 2022 [cited 2024 Oct 29]; Available from: https://www.frontiersin.org/journals/digital-health/articles/10.3389/fdgth.2022.1017231/full

17. Were MC, Savai S, Mokaya B, Mbugua S, Ribeka N, Cholli P, et al. mUzima Mobile Electronic Health Record (EHR) System: Development and Implementation at Scale. J Med Internet Res [Internet]. 2021 Dec 14 [cited 2024 Oct 29];23(12):e26381. Available from: https://www.jmir.org/2021/12/e26381

18. Awol SM, Birhanu AY, Mekonnen ZA, Gashu KD, Shiferaw AM, Endehabtu BF, et al. Health Professionals’ Readiness and Its Associated Factors to Implement Electronic Medical Record System in Four Selected Primary Hospitals in Ethiopia. Adv Med Educ Pract [Internet]. 2020 Feb [cited 2024 Oct 29]; Volume 11:147–54. Available from: https://www.dovepress.com/health-professionals-readiness-and-its-associated-factors-to-implement-peer-reviewed-article-AMEP

19. Ogundaini O, de la Harpe R, McLean N. Unintended consequences of technology-enabled work activities experienced by healthcare professionals in tertiary hospitals of sub-Saharan Africa. African Journal of Science, Technology, Innovation and Development [Internet]. 2022 [cited 2024 Oct 29];14(4):876–85. Available from: https://doi.org/10.1080/20421338.2021.1899556

20. Ngusie HS, Kassie SY, Chereka AA, Enyew EB. Healthcare providers’ readiness for electronic health record adoption: a cross-sectional study during pre-implementation phase. BMC Health Serv Res [Internet]. 2022 Dec 2 [cited 2024 Oct 29];22(1):282. Available from: https://bmchealthservres.biomedcentral.com/articles/10.1186/s12913-022-07688-x

21. Gumede-Moyo S, Todd J, Bond V, Mee P, Filteau S. A qualitative inquiry into implementing an electronic health record system (SmartCare) for prevention of mother-to-child transmission data in Zambia: a retrospective study. BMJ Open [Internet]. 2019 Sep 1 [cited 2024 Oct 29];9(9):e030428. Available from: https://bmjopen.bmj.com/content/9/9/e030428

22. Sibiya MN, Akinyemi OR, Oladimeji O. Computer Skills and Electronic Health Records (EHRs) in a State Tertiary Hospital in Southwest Nigeria. Epidemiologia 2023, Vol 4, Pages 137-147 [Internet]. 2023 Apr 27 [cited 2024 Nov 17];4(2):137–47. Available from: https://www.mdpi.com/2673-3986/4/2/15/htm

23. Oluoch T, Kwaro D, Ssempijja V, Katana A, Langat P, Okeyo N, et al. Better adherence to pre-antiretroviral therapy guidelines after implementing an electronic medical record system in rural Kenyan HIV clinics: A multicenter pre-post study. International Journal of Infectious Diseases. 2015 Apr 1;33:109–13.

24. Adetoyi OE, Raji OA. Electronic health record design for inclusion in sub-Saharan Africa medical record informatics. Sci Afr [Internet]. 2017 Mar 1 [cited 2024 Oct 29];14(1):10–1. Available from: https://www.sciencedirect.com/science/article/pii/S2468227620300429

25. Rahel Abiy ;  Kassahun Gashu ;  Tarekegn Asemaw ;  Mebratu Mitiku ;  Berhanu Fekadie ;  Zeleke Abebaw ;  Adane Mamuye ;  Ashenafi Tazebew ;  Alemayehu Teklu ;  Fedilu Nurhussien ;  Mihiretu Kebede ;  Fleur Fritz ;  Binyam Tilahun. A comparison of electronic records to paper records in antiretroviral therapy clinic in Ethiopia: what is affecting the quality of the data? Online J Public Health Inform [Internet]. 2018 [cited 2024 Oct 29];10. Available from: https://www.semanticscholar.org/reader/9f8673b847adcc5dc48a286b6d2b9151957be0e6

26. Kiri VA, Ojule AC. Electronic Medical Record Systems: A Pathway to Sustainable Public Health Insurance Schemes in Sub-Saharan Africa. Nigerian Postgraduate Medical Journal [Internet]. 2020 Jan 1 [cited 2024 Oct 29];27(1):1–7. Available from: https://journals.lww.com/npmj/fulltext/2020/27010/electronic_medical_record_systems__a_pathway_to.1.aspx

27. Oluoch T, Katana A, Ssempijja V, Kwaro D, Langat P, Kimanga D, et al. Electronic medical record systems are associated with appropriate placement of HIV patients on antiretroviral therapy in rural health facilities in Kenya: a retrospective pre-post study. Journal of the American Medical Informatics Association [Internet]. 2014 Nov [cited 2024 Oct 29];21(6):1009–14. Available from: https://academic.oup.com/jamia/article-lookup/doi/10.1136/amiajnl-2013-002447

28. Odekunle FF, Srinivasan S, Odekunle RO. Why Sub-Saharan Africa Lags in Electronic Health Record (EHR) Adoption and Possible Strategies to Increase EHR Adoption in this Region. Journal of Health Informatics in Africa [Internet]. 2018 Nov 4 [cited 2024 Oct 29];5(1):8–15. Available from: https://www.jhia-online.org/index.php/jhia/article/view/147

29. Muinga N, Magare S, Monda J, Kamau O, Houston S, Fraser H, et al. Implementing an Open Source Electronic Health Record System in Kenyan Health Care Facilities: Case Study. JMIR Med Inform [Internet]. 2018 Apr 18 [cited 2024 Oct 29];6(2):e22. Available from: http://medinform.jmir.org/2018/2/e22/

30. Yehualashet G, Andualem M, Tilahun B. The attitude towards and use of electronic medical record system by health professionals at a referral hospital in northern Ethiopia: Cross-sectional study. J Health Informatics in Africa. 2015;3(1):25–37.
